# Anti-Termitic Activity of Three Plant Extracts, Chlorpyrifos, and a Bioagent Compound (Protecto) against Termite *Microcerotermes eugnathus* Silvestri (Blattodea: Termitidae) in Egypt

**DOI:** 10.3390/insects11110756

**Published:** 2020-11-04

**Authors:** Mohamed Z. M. Salem, Mona F. Ali, Maisa M. A. Mansour, Hayssam M. Ali, Esraa M. Abdel Moneim, Ahmed Abdel-Megeed

**Affiliations:** 1 Forestry and Wood Technology Department, Faculty of Agriculture (EL-Shatby), Alexandria University, Alexandria 21545, Egypt; 2 Conservation Department, Faculty of Archaeology, Cairo University, Giza 12613, Egypt; monalyeg@yahoo.com (M.F.A.); maisamansour_40@yahoo.com (M.M.A.M.); 3 Botany and Microbiology Department, College of Science, King Saud University, P.O. Box 2455, Riyadh 11451, Saudi Arabia; hayhassan@ksu.edu.sa; 4 Timber Trees Research Department, Sabahia Horticulture Research Station, Horticulture Research Institute, Agriculture Research Center, Alexandria 21526, Egypt; 5 Conservation Department, Ministry of Antiquities, Giza 12578, Egypt; esraamahmoud822@yahoo.com; 6 Department of Plant Protection, Faculty of Agriculture (Saba Basha), Alexandria University, Alexandria 21531, Egypt; hekemdar@yahoo.com

**Keywords:** Bir al-Shaghala cemeteries, plant extracts, chemical pesticide, bioagent pesticide, termite

## Abstract

**Simple Summary:**

The termite *Microcerotermes eugnathus* Silvestri (Blattodea, Termitidae) showed structural damage in Bir al-Shaghala cemeteries located in the oasis of Dakhla, Egypt. The mud tubes of this termite spread inside and over the mural painted floors of the tombs. Extracts from *Lavandula latifolia*, *Origanum vulgare*, and *Syzygium aromaticum* were tested for their anti-termitic activity and compared with the bio-insecticide, *Bacillus thuringiensis var. kurstaki* (Protecto 9.4% WP) and Dursban (Chlorpyrifos 48%). The bioassay experimental showed that the extracts have low activity against *M. eugnathus* compared to Protecto and Dursban, but the extract from *O. vulgare* showed promising natural termiticides.

**Abstract:**

A trend towards environmentally friendly chemicals for use in termite management has been occurring globally. This study examined three naturally occurring plant extracts from *Lavandula latifolia* (Spike lavender)*, Origanum vulgare* (Marjorum), and *Syzygium aromaticum* (Clove) against the termite *Microcerotermes eugnathus*. Plant extract results were compared to two commercially used termite pesticides, the bio-insecticide, *Bacillus thuringiensis var. kurstaki* (Protecto 9.4% WP) and Dursban (Chlorpyrifos 48%). Gas chromatography–mass spectrometry (GC-MS) analysis was used to identify the main compounds in the three plant extracts. The main compounds in *Lavandula Latifolia* were linalool (21.49%), lavandulol (12.77%), *β*-terpinyl acetate (10.49%), and camphor (9.30%). *Origanum vulgare* extract contained thymol (14.64%), *m*-cymene (10.63%), linalool (6.75%), and terpinen-4-ol (6.92%) as main compounds. *Syzygium aromaticum* contained eugenol (99.16%) as the most abundant identified compound. The extract of *O. vulgare* caused the highest termite death rate, with an LC_50_ of 770.67 mg/L. Exposure to lavender extract showed a high death rate with an LC_50_ of 1086.39 mg/L. Clove extract did not show significant insecticidal activity with an LC_50_ > 2000 mg/L. Significant termiticide effects were found, with LC_50_ values of 84.09 and 269.98 mg/L for soldiers and workers under the application of Dursban and Protecto, respectively. The LC_50_ values reported for nymphs were <120, <164.5, and 627.87 mg/L after exposure to Dursban, Protecto, and *O. vulgare* extract, respectively. The results of the study show that some of the extracts have low toxicity compared to the bioagent and Dursban, and may show promise as natural termiticides, particularly as extracts from *O. vulgare*.

## 1. Introduction

**Figure 1:**
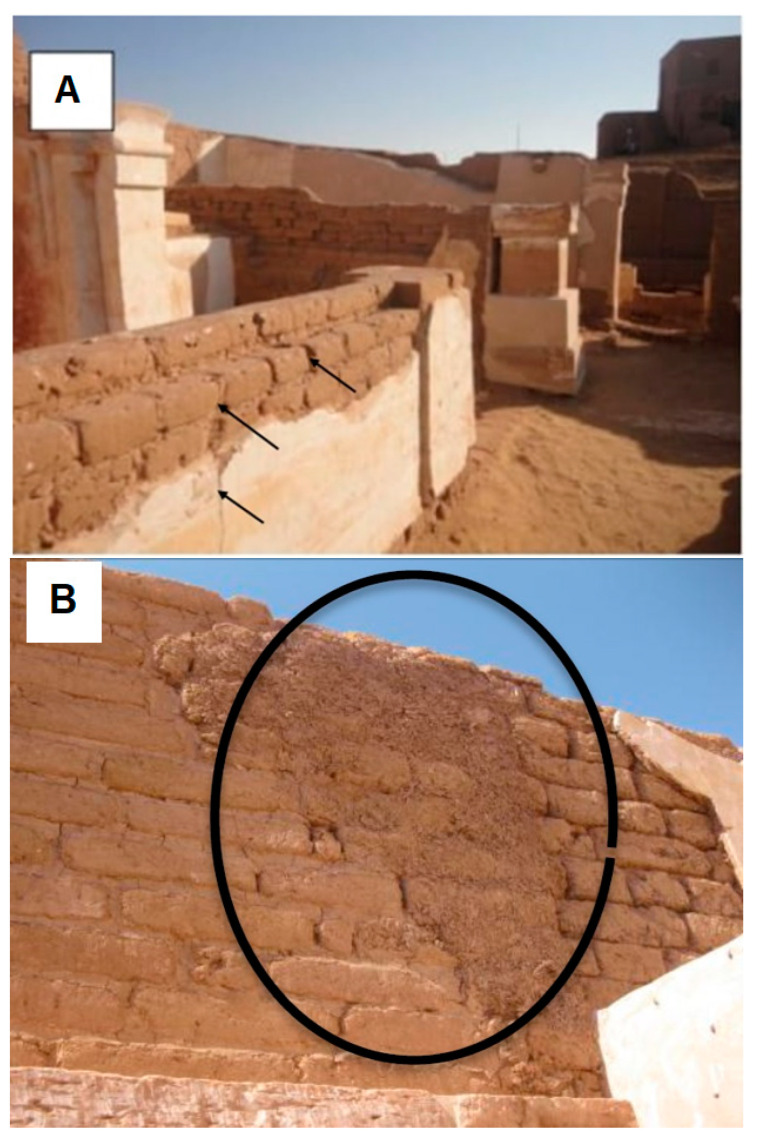
Bir Al-Shaghala Cemetery views and damage caused by termite attack. (**A**) damage occurred in the binding materials of mudbricks; (**B**) damage to the surface walls and shelter tubes of termites over the mudbricks.

Termites, although important in ecosystems, are economically important structural pests to wood and cellulosic material in service [1,2,3,4,5,6]. Bir al-Shaghala cemeteries, located to the west of the city of Mut, 3 km from *Dakhla Oasis*, Egypt, are the site of heavy infestation by the termite *Microcerotermes eugnathus* Silvestri (Blattodea, Termitidae) [7,8,9,10]. These termites forage and feed on the cellulosic material used in the construction of the mud brick from which the tombs are built. Termites infest the walls and tomb foundations, building noticeable foraging tubes (Figure 1).

In Egypt, this termite was reported along the northwestern coast [9] and in the Western Desert where the oasis of Dakhla is located [7]. Termite mud tubes spread inside and over the mural painted floors of the tombs, causing structural damage that often needs repair. The damage observed in some of the monastery buildings, as well as in a church located in a nearby village of Saint Catherine, was associated with *M. eugnathus* [8].

Treatments used to control this termite include bioagents, chemical pesticides, and natural products. Natural products investigated for termite management include plant extracts. In many parts of the world, chemical pesticides are considered poor for the environment and new research is examining the use of naturally occurring, less environmentally toxic, compounds [11,12,13].

Bioagents such as *Bacillus thuringiensis var. kurstaki* and *Stenotrophomonas maltophilia* Palleroni & Bradbury have potential application as biological control agents of termites [14,15,16]. The entomopathogenic bacteria *B. thuringiensis* is an example of this [17,18]. Chitinase produced by *S. maltophilia* has shown potential application as a biocontrol agent and could degrade chitin in the termites, *Coptotermes heimi* (Wasmann) (Blattodea: Rhinotermitidae) and *Heterotermes indicola* (Wasmann) (Blattodea: Rhinotermitidae) [15].

Dursban (Chlorpyrifos 48%) is an organophosphate pesticide that acts on the insect nervous system by inhibiting acetylcholinesterase (AChE) [19,20,21]. Dursban is still used in the Egyptian agriculture ecosystem, with some conditions [22]. Starting from May 2019, the California Department of Pesticide Regulation said, “the registration that allows Dursban to be sold in California will be canceled”, but this process could take up to two years [23]. In 2016, Denmark, Finland, Germany, Latvia, Sweden, Ireland, Lithuania, Slovenia and the United Kingdom banned, or never authorized, the use of Dursban products, and the Swiss government decided to withdraw permissions for 12 Dursban and Dursban-methyl products according to Tagblatt [24]. As toxic Dursban has been withdrawn for use in many countries, less toxic alternatives are being examined.

Natural products are being studied as potential termiticides. The termite *C. intermedius* Silvestri (Blattodea: Rhinotermitidae) died within 30, 40, and 110 min when exposed to 70% ethanol, aqueous, and acetone extracts from seeds of *Parkia biglobosa* (Jacq) Benth, respectively, at the concentration of 4 gm/L [25]. Seed oils from *Khaya senegalensis* (Desr.) A. Juss., *K. ivorensis* A. Chev. and *Swietenia mahagoni* (L.) Jacq. showed potential insecticidal efficacy towards the dry wood termite *Kalotermes flavicollis* (Fabricius, 1793) (Blattodea: Kalotermitidae) [26]. Water extracts from *Larix leptolepis* (Lamb.) Carr. Wood, with its large quantities of flavonoids, showed strong feeding deterrent activity against the subterranean termite *Coptotermes formosanus* Shiraki (Insecta: Blattodea: Rhinotermitidae) [27]. *Jatropha curcas* L. oil showed maximum wood protection against *Microcerotermes beesoni* Snyder (Termitidae: Amitermitinae) at a concentration of 20% [28].

Some heartwoods show resistance to termite attack, which is attributed to the presence of toxic compounds within the heartwood [29,30]. For example, heartwood extractives from *Tectona grandis* L.f., *Dalbergia sissoo* Roxb., *Cedrus deodara* (Roxb.) G.Don and *Pinus roxburghii* Sarg. rapidly lowered numbers of protozoans in the hindgut of *Reticulitermes flavipes* (Kollar) (Insecta: Blattodea: Rhinotermitidae) and *H. indicola* workers, which were closely correlated with the mortality of worker [30]. Wood samples from southern pine and poplar impregnated with the extractives from *Morus alba* L. heartwood resulted in 100% mortality in *R. flavipes* after feeding [31]. Methanolic and ethyl acetate extracts were found to be to be effective against the termite *Microcerotermes beesoni* Snyder (Termitidae: Amitermitinae) [32]. Heartwood extract from of *D. sissoo* showed termite mortality at 10 mg/mL against *H. indicola* and *R. flavipes* with LC_50s_ at 5.54 and 3.89 mg/mL, respectively [33]. *H. indicola* mortality reached more than 75% after feeding on *Populus deltoides* W.Bartram ex Marshall wood treated with extract from *M. alba* [34]. 

Many plants contain bioactive compounds such as phenols, terpenoids (monoterpenes, sesquiterpenes, diterpenes, sesterpenes, and triterpenes), aldehydes, ketones, and other compounds that have a strong effect against microorganisms and termites [35,36,37,38,39,40,41]. For example, extracts from *Cymbopogon citratus* (DC.) Stapf, *Eucalyptus globulus* Labill., *Syzygium aromaticum* (L.) Merr. and L. M. Perry, *Origanum vulgare* L., *Rosmarinus officinalis* L., *Cinnamomum verum* J.Presl, and *Thymus vulgaris* L. showed strong termiticidal activity against *Odontotermes assamensis* Holmgren (Blattodea: Termitidae) [42]. Insecticidal activity from *Cananga odorata* (Lam.) Hook.f. and Thomson flower extract of 2 mg/filter paper showed cumulative mortalities of 18% and 94%, after 2 and 7 days of exposure, respectively, against the termite *Reticulitermes speratus* (Kolbe) (Rhinotermitidae: Blattodea) [43]. *K. flavicollis* pseudergates fed *Casuarina* sp. wood wafers, previously treated separately with *Taxodium distichum* (L.) Rich., *E. citriodora* Hook. and *Cupressus sempervirens* L. extracts, showed reduced numbers and vigour in spirochaete and flagellate populations [44]. At a dose of 6 μL/Petri-plate, *Tagetes erecta* L. leaf extract, caused 100% mortality in *Odontotermes obesus* (Rambur) (Blattodea: Termitidae) after 24 h of exposure [45]. Therefore, in the present study, three plant extracts were used as natural products to control the termite *Microcerotermes eugnathus*.

Lavender (*Lavandula latifolia* Medik.) belongs to the family Lamiaceae and is native to the Mediterranean. It is also cultivated in different regions of the world. The bioactivity of *L. angustifolia* Mill. and *L. latifolia* extracts from Spain were found to be associated with the presence of linalool, camphor, *p*-cymene, and limonene [46]. Linalool and linalyl acetate have been shown to be the main compounds in the extract of fresh and dry flowers from lavender [47]. Other constituents of lavender include *α*-pinene, thujene, *β*-pinene, sabinene, myrcene, *p*-cymene, limonene, camphene, camphor, 1,8-cineole, (*Z*)- and (*E*) ocimene, terpinene, terpinene-4-ol, lavandulol, lavandulylacetate, *β*-caryophyllene, and (*Z*)- and (*E*) farnesene [48]. 

Several *Origanum* (Marjoram–Lamiaceae family) species are characterized by the presence of two main chemotypes, thymol and carvacrol, while others have *γ*-terpinene, *p*-cymene, *cis*-sabinene hydrate, terpinen-4-ol, *α*-terpineol, *α*-terpinene, *γ*-terpinene, sabinene, and linalool as their main compounds [39,49,50,51,52]. *O. vulgare* L. ssp. *vulgare* extract showed the presence of caryophyllene, spathulenol, germacrene-D, and α-terpineol and had antimicrobial properties [53]. Terpinen-4-ol, *γ*-terpinene, *α*-terpineol, thymol, carvacrol, *cis*-sabinene-hydrate, linalool, p-cimene, 1,8-cineole were identified as the main compounds in the extract of *O. vulgare* L. [54]. *O. vulgare* extract and its major compounds carvacrol, p-cymene, and *γ*-terpinene showed potent larvicidal activity against the cotton bollworm, *Helicoverpa armigera* (Hübner) (Lepidoptera: Noctuidae: Heliothinae) [55]. 

Clove (*Syzygium aromaticum* (L.) Merr. and L.M.Perry, Myrtaceae family) bud extract has shown the presence of eugenol, eugenol acetate and β-caryophyllene as main compounds with percentages of 88.61, 8.89 and 1.89%, respectively. It exhibited strong contact toxicity against *Cacopsylla chinensis* (Yang and Li) (Hemiptera: Psyllidae) with LD_50_ values of 0.730 µg/adult and 1.795 µg/nymph, respectively [56]. *S. aromaticum* extract gave LC_50_ values of 13.871 and 15.551 μL against larvae and adult *Tribolium castaneum* (Herbst) (Coleoptera: Tenebrionidae) 48 h after fumigation [57]. Other studies have indicated that the clove extract caused 100% mortality 48 h after treatment with the concentrations of 17.9 and 35 μL/g, and with LC_50_ values of 9.45 and 10.15 μL/g, against *Acanthoscelides obtectus* (Say) (Coleoptera: Chrysomelidae: Bruchinae) and *Sitophilus zeamais* Motschulsky (Coleoptera: Curculionidae), respectively [58]. 

This research aimed to (1) determine the chemical composition of three plant extracts (lavender, marjoram and clove) with potential anti-termitic activities; (2) evaluate the anti-termitic activity of the three plant extracts against *Microcerotermes eugnathus* compared with two commercial pesticides used in termite control; the bioagent “*Bacillus thuringiensis var. kurstaki*” (Protecto 9.4% WP) and the chemical compound Dursban (chlorpyrifos 48%). In addition, the chemical composition of the three extracts were analyzed with using GC-MS apparatus.

## 2. Materials and Methods

### 2.1. Termite Infestation in the Cemeteries of Bir al-Shaghala at the Oases of Dakhla

Termite infestation in the cemeteries of Bir al-Shaghala located at the oases of Dakhla, Egypt was noted by the presence of *Microcerotermes eugnathus* foraging tubes. Termite tunnels could be observed on the floors and tomb substructure, causing cracks and separation of mortar. This termite species produces winged alates during the months of April and January, the remnants of which (wings) could often be observed around the tombs [59]. Figure 1A,B shows the cemetery termite damage. 

### 2.2. Plant Extracts

Three plant extracts from Spike Lavender (*Lavandula latifolia* Medik., Lamiaceae) dry flower, Marjoram (*Origanum vulgare* L., Lamiaceae) dry leaves and Clove (*Syzygium aromaticum* (L.) Merrill and Perry, Mytaceae) dry buds were selected for their potential effect on termites. The extraction process was carried out at the National Research Center in Cairo, Egypt. Plant material was shade air-dried in the laboratory at room temperature for one week, then ground into a fine powder in an electrical blender. About 100 g of the fine powdered material was put into a 2-L flask containing 1000 mL of distilled water (DW) and extracted by the hydrodistillation method using a Clevenger-type apparatus for 3 h [60]. 

### 2.3. Gas Chromatography–Mass Spectrometry (GC-MS) Analysis of Extracts

The chemical composition of extracts from *L. latifolia, O. vulgare*, and *S. aromaticum* were determined using a Trace GC-TSQ Evo 9000 mass spectrometer (Thermo Scientific, Austin, TX, USA) with a TG–5MS direct capillary column (30 m × 0.25 mm × 0.25 µm film thickness). Extracts were diluted in n-hexane solvent in the ratio of 3:1 (3 n-heaxane: 1 extract sample) before being injected to the GC-MS. The column oven temperature was initially held at 50 °C and then increased by 5 °C/min to 250 °C and held for 2 min, then increased to a final temperature of 310 °C by 25 °C/min and held for 2 min. The injector and MS transfer line temperatures were kept at 270 and 260 °C, respectively; helium was used as a carrier gas at a constant flow rate of 1 mL/min. The solvent delay was 3 min, and diluted samples of 2 µL were injected automatically using an Autosampler AS1310 coupled with GC in the splitless mode. EI mass spectra were collected at 70 eV ionization voltages over the *m/z* range of 50–650 in full scan mode. The ion source temperature was set at 250 °C. The components were identified by comparison of their retention times and mass spectra with those in the WILEY 09 and NIST 11 mass spectral databases [61]. The match factor (MF) between the mass spectrum obtained for each compound and the library mass spectra for each compound was measured and reported; it was accepted if its value was more than or equal to 650 [36,38,39,40,62,63].

### 2.4. Preparation of the Plant Extracts, Protecto and Dursban

Extract concentrations for laboratory testing were prepared by mixing with 0.01% of Tween 80 and diluting with distilled water to obtain the following concentrations: 125, 250, 500, 1000, and 2000 mg/L. The bioagent “*Bacillus thuringiensis var. kurstaki*” (Protecto 9.4% WP) was diluted in distilled water to obtain the following concentrations: 164.5, 258.5, 376, 756, and 1410 mg/L. Dursban (Chlorpyrifos 48%) was diluted in distilled water and prepared in concentrations of 120, 240, 360, 480, and 960 mg/L.

### 2.5. Collecting and Rearing the Termites 

*Microcerotermes eugnathus* castes were collected from soil around cemeteries and kept in a container with a portion of termite nesting material. Briefly, from the experimental location, termites were placed inside a special box (4-L in capacity) for preservation and the box was covered with wool textile to maintain the temperature, because the temperatures in the interior oases are as high as 50 °C. During the transfer to the laboratory in Cairo, the death of the termites occurs due to the different environmental conditions, so it was necessary to warm the termites. Termites’ death was also observed during transportation to the laboratory or even in the laboratory during the preparation for the laboratory experiment. Therefore, 10% glucose sugar solution [64,65] was placed where sterile cotton was absorbed into the sugar solution and suspended in a flask above the cardboard feeding medium as a source of nourishment for termites, to give moisture and nutrition. The termites were incubated at 48 ± 3 °C in order to provide a medium close to that of the oasis.

### 2.6. Testing Anti-Termitic Activity (No-Choice Bioassay Method)

A no-choice bioassay method [66,67] was employed to measure the anti-termitic activity of the three plant extracts, Protecto, and Dursban. 1125 pieces of cardboard similar to the laboratory rearing material were prepared and divided into three groups and each group contained 375 pieces and divided to five subgroups (75 pieces). In the first group, each subgroup (75 pieces) was dipped in each of the concentrations of plant extracts, in the second group (375 pieces); each subgroup (75 pieces) was dipped in each of the concentrations of Protecto, and the third group (375 pieces) each subgroup (75 pieces) was dipped in in each of the concentrations of Dursban. Treated cardboard samples were placed in glass flasks (2-L capacity), 15 pieces were used for each concentration, where termites were added and the flasks were sealed using a piece of gauze to allow ventilation. Each flask contained 10 workers and five soldiers (Total 15). With the same manner, the experiment was carried out with nymphs, where each flask contained five nymphs.

Untreated control replicates (five replicates) contained 10 workers, five soldiers and five nymphs, who were supplied with pieces of untreated cardboard paper only. Each concentration tested was replicated five times. All the treatments were kept in darkness at 48 ± 3 °C. After seven days, the test was halted, the number of surviving termites was counted, and percent mortality was calculated. The lethal concentration (LC_50_) expressed as mg/L was calculated from log-concentration mortality regression lines [38].

### 2.7. Statistical Analysis 

Probit analysis was used to estimate the lethal concentrations LC_50_ from mortality data, and the slope was obtained according to Finney [68].

## 3. Results and Discussion 

### 3.1. Chemical Composition of Three Plant Extracts

Table 1 shows the results of the Gas chromatography–mass spectrometry (GC-MS) analysis of *L. latifolia* extract. The main constituents, were linalool (21.49%), lavandulol (12.77%), *β*-terpinyl acetate (10.49%), camphor (9.30%), linalyl acetate (7.87%), (−)-*β*-fenchol (5.12%), isobornyl acetate (5.00%), *α*-pinene (3.81%), terpineol (3.64%), and L-*α*-terpineol (2.62%).

Table 2 shows the results of the GC-MS analysis of extract from *O. vulgare* dry leaves. The main components were thymol (14.64%), *m*-cymene (10.63%), terpinen-4-ol (6.92%), linalool (6.75%), estragole (5.42%), *γ*-terpinene (5.39%), anethole (5.00%), eucalyptol (3.61%), borneol (3.29%), *β*-caryophyllene (3.29%), *α*-terpinene (2.89%), carvacrol (2.58%), camphor (2.36%) and carvacryl methyl ether (2.34%). 

Table 3 shows results of the GC-MS analysis of *S. aromaticum* extract. The main component was eugenol (99.16%).

### 3.2. Anti-Termitic Activity Comparison of Plant Extracts, Protecto and Dursban against Microcerotermes Eugnathus

The effects of the tested plant extracts showed that marjoram extract had the highest toxicity against *Microcerotermes eugnathus*. After a seven-day exposure to this extract, the LC_50_ was 770.67 mg/L against soldiers and workers and 627.87 mg/L against nymphs. Lavender extract had less of toxic effect against *M. eugnathus* than marjoram, with an LC_50_ of 1086.39 mg/L for the group of soldiers and workers and 1140.74 mg/L for nymphs. Clove extract showed the lowest insecticidal activity against *M. eugnathus* for both nymphs and soldier/workers, with an LC_50_ > 2000 mg/L (Table 4). Marjoram extract was most effective as an insecticide against *M. eugnathus*. Table 4 shows that the LC_50_ values for Dursban for individuals and nymphs were 84.09 and <120 mg/L, respectively. LC_50_ values for individuals and nymphs were <164.5 and 269.98 mg/L for Protecto, respectively. 

Our results show that Dursban caused the highest termite mortality of 80%, while Protecto ranked second at 75% mortality. For the plant extracts, marjoram caused the highest mortality at 70% (Table 5).

## 4. Discussion

Many plant compounds can incur high mortality in termites. This is related to their mechanism of action on the insect nervous system [69]. Plant compounds may also have neurotoxic (hyperactivity, seizures, and tremors) modes of action [70,71,72]. The data presented in this work clearly show big differences in chemical components among plant samples depending on their origin, even in the same species.

Earlier studies showed that linalool, eucalyptol, camphor, α-pinene, α-terpineol, and α-bisabolene were the most abundant components in *L. latifolia* extract grown in the southeast of Spain [47]. Another study found linalool, 1,8-cineole, and camphor were dominant in extracts from seven natural populations of *L. latifolia* from the eastern Iberian Peninsula [73] and from wild Spanish populations [74]. The chemical composition of lab-distilled extract from *L. latifolia* and commercial spike lavender extract characterized 1.8-cineole, linalool, and camphor as major constituents [75]. Compounds from fresh stems and dry stemless flowers of lavender cultivars in Turkey showed linalool, linalyl acetate, camphor, sabinene, cimene, and terpeneol-4-ol as the main compounds [76]. Others reported *α*-fenchone, myrtenyl acetate, α-pinene, camphor and 1,8-cineole in the flowers of *L. stoechas* L. ssp. *stoechas* [77]. Fenchone and camphor (68.2% and 11.2%, respectively) were found in extract of *L. stoechas* from Tunisia [78].

Linalool, borneol and terpinene-4-ol, 1,8-cineole, camphor, and linalyl acetate were reported in lavandin (*Lavandula x intermedia* Emeric ex Loisel.) extract from Macedonia [79]. In another study from Iran, extract of dried lavender consisted predominately of 1,8-cineole, linalool, borneol, camphor, and linalyl acetate [80]. Linalool, camphor, 1,8-cineole, lavandulol, *p*-cymen-8-ol and bornyl acetate with values of 32.3%, 12.4%, 11.7%, 8.7%, 7.7%, and 4.2%, respectively, were the main compounds in *L. latifolia* extract from the Northern Tunisia [81]. Chemotypes of *L. latifolia* extracts from Spain, characterized by 1,8-cineole with high amounts (36.3–33.65%) [75,82], while *L. latifolia* from Iran showed the presence of linalool (31.9–30.6%) as the main compound [47]. In other species of *Lavandula*, 1,8-cineole, 2-*β*-pinene, *α*-thujone, camphor and *cis-α*-bisabolene were the main compounds isolated from *L. angustifolia* extract [83].

Previous work identified *α*-terpinene, *γ*-terpinene, *cis*-sabinene-hydrate, linalool, terpinen-4-ol, terpineol, thymol, and carvacrol as the main compounds in *O. vulgare* extract with percentages of 5.58, 12.32, 3.35, 3.47, 21.43, 3.17, 9.45, and 11.67%, respectively [54]. Other work found that *O. vulgare* extract contained *p*-cymene, linalool, thymol, caryophyllene, and carvacrol. These authors showed insecticidal activity against *Schistocerca gregaria* (Forskål) (Orthoptera: Acrididae) [84]. In the present study, thymol was found at 14.64% in *O. vulgare* extract. Other studies reported this same compound at 9.45% [54], 44.55% [85], 26.75% [86], and 58.31% [87]. After 5 h exposure, *Coleus amboinicus* Lour. extract caused 100% mortality to the termite *Odontotermes obesus* at a dose of 2.5 × 10^−2^ mg/cm^3^. This study found that thymol was the main compound, with 94.3% [88]. Extracts from *Pittosporum undulatum* Vent., *Lippia sidoides* Cham. and *Lippia gracilis* Schauer showed 100% mortality to the termite *Heterotermes sulcatus* Mathews (Blattodea: Rhinotermitidae) after 14 days of exposure, where the activity was related to the major compounds, limonene (80.8%), carvacrol (45.6%) and thymol (78.55%), that were identified in each extract, respectively [69].

Terpineol (22.85%), α–terpinene (20.60%), caryophyllene (6.75%) and thymol (4.53%) were the main compounds isolated from *O. vulgare* extract [89]. *O. vulgare* extract with its main compounds of thymol (40.35%), *p*-cymene (17.32%), *γ*-terpinene (15.66%), and carvacrol (12.15%), observed 72% and 88% mortality at 48 and 72 h, respectively, against *Sitophilus oryzae* (L.) (Coleoptera: Curculionidae) [90]. *O. vulgare* extract showed toxicity in higher doses against *Plutella xylustella* L. (Lepidoptera: Pyralidae) [91]. After application, *O. vulgare* extract at 40% exhibited 100% repellency against *Cimex lectularius* Linnaeus (Hemiptera: Cimicidae) [89].

Oxygenated monoterpenes such as thymol, eucalyptol, linalool and carvacrol found in the extracts from the present study are reported to be more toxic against termite workers [92]. For example, thymol and carvacrol from *Origanum* were shown to have insecticidal activity against larvae of *Culex pipiens* L. (Diptera: Culicidae) [93]. The extract of *Lippia sidoides* contained 44.55% thymol and showed high insecticidal activity against the termite *Nasutitermes corniger* (Blattodea: Termitidae) with 48 h exposure at a dose of 0.27 μg/mg [85].

In this study, clove extract showed the lowest activity against *Microcerotermes eugnathus* with an LC_50_ value of >2000 mg/L. Eugenol has been shown to be an effective fumigant and feeding deterrent against the termite *C. formosanus* [94]. Carvacrol has been shown to produce potent, acute toxicity to insects, nematodes, and mites [95]. It causes positive allosteric modulators in insects to bind to *γ*-aminobutyric acid, causing inhibitory symptoms in the nervous system [3]. Carvacrol caused 100% mortality of *Odontotermes assamensis* Holmgren (Blattodea: Termitidae) at 2.5 mg/g after 8 days [42]. At 1.5 mg/g and after 48 h, it exhibited anti-termitic activity against *Reticulitermes messperatus* Kolbe (Blattodea: Rhinotermitidae) nymphs [96,97]. *S. aromaticum* extract at 0.5 mL/L after 10 min of exposure showed 100% mortality against *Coptotermes formosanus* Shiraki (Insecta: Blattodea: Rhinotermitidae) [98]. Clove bud extract caused 100% mortality at 0.5 μL/L of air by fumigation method against *R. speratus* [99].

Our present results show a higher resistance of *M. eugnathus* against termiticides than those of other termites. In addition, our results suggest that *O. vulgare* extract caused moderate activity against the termite *M. eugnathus* compared to commercial termiticides tested. Further research may show that *O. vulgare* extract or its derivatives could potentially be used to control and manage some termite infestations and lessen or limit the amount of more toxic pesticides currently in use. 

## 5. Conclusions

Chemical pesticides had the greatest impact on the mortality of the termite *M. eugnathus*. However, due to the highly toxic nature of Dursban and the fact that it has been withdrawn for use in some countries, the search for less toxic and natural termiticides from plants is of interest. Extract from the plant *Origanum vulgare* showed some promise as a plant-based toxicant for this termite. 

## Figures and Tables

**Table 1:** Gas chromatography–mass spectrometry (GC-MS) results for the extract of *Lavandula latifolia*.

Retention Time (min)	Percentage in the Oil (%)	Compound Name	Match Factor (MF)
4.74	3.81	*α*-Pinene	938
6.95	10.49	*β*-Terpinyl acetate	913
8.95	21.49	Linalool	944
10.48	9.30	Camphor	946
11.56	2.62	L*-α*-Terpineol	938
11.70	3.64	*α*-Terpineol	940
11.85	5.12	(−)-*β*-Fenchol	935
13.29	12.77	Lavandulol	938
13.72	3.32	Linalyl anthranilate	914
14.35	5.00	Isobornyl acetate	953
16.23	7.87	Linalyl acetate	929

**Table 2:** GC-MS results for the extract of *Origanum vulgare*.

Retention Time (min)	Percentage in the Oil (%)	Compound Name	Match Factor (MF)
6.61	2.89	*α*-Terpinene	939
6.85	10.63	*m*-Cymene	939
7.04	3.61	Eucalyptol	939
7.75	5.39	*γ*-Terpinene	939
8.92	6.75	Linalool	930
9.98	2.36	Camphor	955
10.64	3.29	Borneol	937
11.00	6.92	Terpinen-4-ol (4-Terpinenol)	943
11.58	5.42	Estragole (4-Allylanisole)	956
12.77	2.34	Carvacryl methyl ether	873
13.95	5.00	Anethole	955
14.31	14.64	Thymol	929
14.55	2.58	Carvacrol	930
17.44	3.29	*β*-Caryophyllene	954

**Table 3:** GC-MS results for the extract of *Syzygium aromaticum*.

Retention Time (min)	Percentage in the Oil (%)	Compound Name	Match Factor (MF)
5.92	99.16	Eugenol	852

**Table 4:** The insecticidal activity of three plant extracts, Dursban, and Protecto against termite groups.

Treatment	Termite Groups	LC_50_			LC_90_			Slope ± SE	χ^2^ Sig.
		Conc mg/L	95% CI		Conc mg/L	95% CI			
			Lower	Upper		Lower	Upper		
Spike lavender oil	Nymphs	1140.74	521.93	2493.21	17,591.54	8048.81	38,448.22	1.09 ± 0.17	0.62
	Soldiers and workers	1086.39	563.86	2093.17	10,134.45	5259.96	19,526.21	1.33 ± 0.14	0.88
Marjoram oil	Nymphs	627.87	292.67	1346.97	9474.85	4416.60	20,326.20	1.091 ± 0.17	0.88
	Soldiers and workers	770.67	420.41	1412.73	7006.47	3822.17	12,843.68	1.42 ± 0.13	0.73
Clove oil	Nymphs	>2000							
	Soldiers and workers	>2000							
Dursban	Nymphs	<120							
	Soldiers and workers	84.09	45.260	156.255	568.587	306.012	1056.464	1.55 ± 0.13	0.99
Protecto	Nymphs	<164.5							
	Soldiers and workers	269.98	108.85	669.67	7163.84	2888.22	17,768.94	0.91 ± 0.21	0.99

**Table 5:** Effect of various compounds on the mortality of the termite *Microcerotermes eugnathus* (soldiers/workers and Nymphs) in a no-choice laboratory test.

Dursban		Protecto		Plant Extracts			
Conc (mg/L)	Mortality %	Conc (mg/L)	Mortality %	Conc (mg/L)	Mortality (%)		
					Marjoram	Lavender	Clove
Control	0	Control	0	Control	0	0	0
120	48	164.5	35	125	15	10	15
240	51	258.5	45	250	20	15	20
360	60	376	51	500	20	15	20
480	65	756	50	1000	30	40	20
960	80	1410	75	2000	70	50	25
